# Omalizumab Is Equally Effective in Persistent Allergic Oral Corticosteroid-Dependent Asthma Caused by Either Seasonal or Perennial Allergens: A Pilot Study

**DOI:** 10.3390/ijms18030521

**Published:** 2017-02-28

**Authors:** Christian Domingo, Xavier Pomares, Albert Navarro, Núria Rudi, Ana Sogo, Ignacio Dávila, Rosa M. Mirapeix

**Affiliations:** 1 Pulmonary Service, Corporació Sanitària Parc Taulí, Parc Taulí, 1, 08208 Sabadell, Barcelona, Spain; jpomares@tauli.cat (X.P.); asogo@tauli.cat (A.S.); 2 Department of Medicine, Faculty of Medicine, Universitat Autònoma de Barcelona (UAB), Av. Can Domènech s/n, 08193 Bellaterra, Barcelona, Spain; 3 CIBER de Enfermedades Respiratorias, CIBERES, Madrid, Spain; 4 Biostatistics Unit, Faculty of Medicine, Universitat Autònoma de Barcelona (UAB), Av. Can Domènech s/n, 08193 Bellaterra, Barcelona, Spain; Albert.navarro@uab.cat; 5 Department of Pharmacy, Corporació Sanitària Parc Taulí, Parc Taulí, 1, 08208 Sabadell, Barcelona, Spain; nrudi@tauli.cat; 6 Allergy Service, University Hospital of Salamanca, Biomedical and diagnostics sciences, Universidad de Salamanca, 37008 Salamanca, Spain; idg@usal.es; 7 Department of Morphological Sciences, Faculty of Medicine, Universitat Autònoma de Barcelona (UAB), Av. Can Domènech s/n, 08193 Bellaterra, Barcelona, Spain; Rosa.mirapeix@uab.cat

**Keywords:** severe allergic asthma, seasonal, perennial, omalizumab, pathophysiology

## Abstract

Omalizumab is marketed for chronic severe asthma patients who are allergic to perennial allergens. Our purpose was to investigate whether omalizumab is also effective in persistent severe asthma due to seasonal allergens. Thirty patients with oral corticosteroid-dependent asthma were treated with Omalizumab according to the dosing table. For each patient with asthma due to seasonal allergens, we recruited the next two consecutive patients with asthma due to perennial allergens. The dose of oral methyl prednisolone (MP) was tapered at a rate of 2 mg every two weeks after the start of treatment with omalizumab depending on tolerance. At each monthly visit, a forced spirometry and fractional exhaled nitric oxide (FeNO) measurement were performed and the accumulated monthly MP dose was calculated. At entry, there were no differences between groups in terms of gender, body mass index or obesity, year exacerbation rate, monthly dose of MP, FeNO and blood immunoglobuline E (IgE) values, or spirometry (perennial: FVC: 76%; FEV_1_: 62%; seasonal: FVC: 79%; FEV_1_: 70%). The follow-up lasted 76 weeks. One patient in each group was considered a non-responder. Spirometry did not worsen in either group. There was a significant intragroup reduction in annual exacerbation rate and MP consumption but no differences were detected in the intergroup comparison. Omalizumab offered the same clinical benefits in the two cohorts regardless of whether the asthma was caused by a seasonal or a perennial allergen. These results strongly suggest that allergens are the trigger in chronic asthma but that it is the persistent exposure to IgE that causes the chronicity.

## 1. Introduction

Omalizumab has been the only monoclonal antibody marketed for treatment of severe allergic asthma for more than 10 years. It was first included in Global Initiative for asthma (GINA) guidelines in November 2006 [1] and became commercially available in Spain in 2007. Initially, the criteria for administering the drug were: age over 12, severe or inadequately controlled allergic asthma due to at least one perennial allergen, maximum weight 150 kg, and blood immunoglobuline E (IgE) concentration between 30 and 700 IU/mL. The maximum monthly calculated dose was 750 mg [2,3]. Since then, age (reduced to six years), IgE concentration (increased to ≥30–1500 kU/L) and total dose of omalizumab (now ≤1200 mg) have been modified, and chronic urticaria has been included as an indication. Omalizumab has shown clinical efficacy and effectiveness in GINA step V asthma patients [4,5,6,7,8,9]. In addition, some studies have shown its efficacy in non-allergic asthma [4,6]. The term “entopy” has been introduced to refer to patients with localized expression of certain allergic characteristics [10] and some authors have proposed that omalizumab may have a wider range of possible prescriptions.

An issue that has never been discussed in the literature is whether omalizumab is equally effective in patients with severe chronic asthma caused by seasonal allergens as in patients with asthma caused by perennial allergens. We designed a pilot study to evaluate whether the effectiveness of omalizumab treatment in chronic severe asthma depends on the perennial or seasonal nature of the allergen. 

## 2. Results

The demographic data at entry are shown in Table 1. Ten patients were included in the seasonal allergen group and 20 in the perennial group. One patient of each group was considered non-responder (in the seasonal group the patient was the one allergic to pollen of *Parietaria*), and so the comparison was made between 9 patients from the seasonal group and 19 from the perennial group. There were no differences in year exacerbation rate, age (perennial: 54 ± 15.8 years; seasonal: 50 ± 17.1; *p* = 0.56) body mass index (BMI), eosinophils, or fractional exhaled nitric oxide (FeNO) (Table 1). Applying the World Health Organization (WHO) classification, there were no differences in overweight (57.9% perennial and 44.4% seasonal; *p* = 0.698) or obesity (36.8% perennial and 11.1% seasonal; *p* = 0.214). IgE concentrations were slightly higher in seasonal allergen patients, although the difference was not statistically significant. Pulmonary function tests (PFTs) were slightly better in the seasonal group (with FEV_1_ around 200 mL higher), although the difference was not statistically significant. The omalizumab dose and methyl-prednisolone (MP) intake were the same in the two groups. The follow-up lasted 76 weeks. The allergen sensitization is described in Table 2.

### 2.1. Changes in the Number of Exacerbations

**Figure 1:**
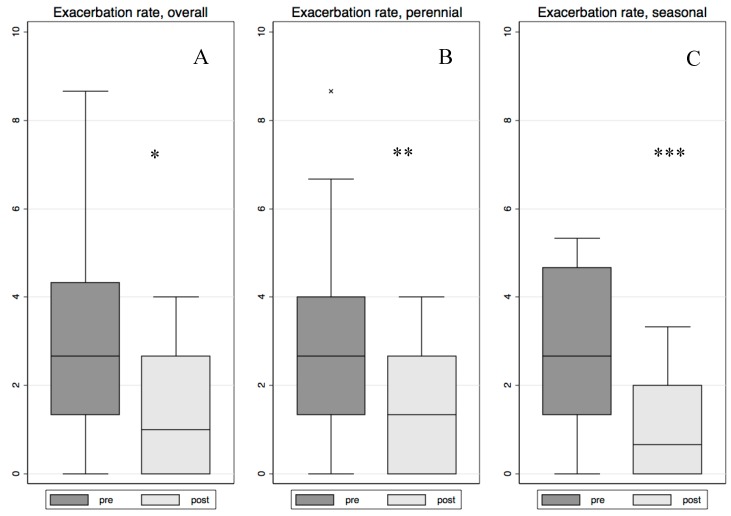
This figure shows the decrease in the year exacerbation rate of the whole group (**A**) (* *p* < 0.001); perennial group (**B**) (** *p* = 0.004) and seasonal group (**C**) (*** *p* = 0.020). **Lower box hinge**: percentile 25. **Upper box hinge**: percentile 75. Line inside the box: percentile 50 (median). Outside value definition: Value lower than *percentile 25 − 1.5 × (percentile 75 − percentile 25)* or value higher than *percentile 75 + 1.5 × (percentile 75 − percentile 25)*. The length of the lower whisker corresponds to the minimum non-outside value and the length of the upper whisker is the maximum non-outside value.

Although we compared the number of exacerbations during the 76 weeks previous to omalizumab treatment with the 76-week follow-up, data are given as annual exacerbation rates. Both groups showed a statistically significant decrease (Figure 1A–C) and no differences were observed between them (*p* = 0.678).

### 2.2. Changes in Oral Corticosteroid Dose

**Figure 2:**
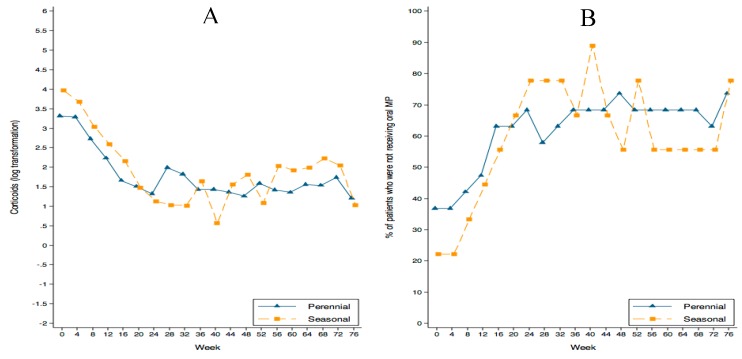
(**A**) Log-transformation of the oral corticosteroid dose decrease. (**B**) The progressive increase in the percentage of patients who were not receiving oral methyl-prednisolone (MP) during the study (*p* < 0.001); there were no differences between groups.

During this period, MP consumption progressively decreased in both groups (*p* < 0.001), but no inter-group differences were found (*p* = 0.856), (Figure 2A). Figure 2B shows the progressive increase in the percentage of patients who were not receiving oral MP during the study (*p* < 0.001); again, no differences were observed between groups (*p* = 0.851). At week 76, mean monthly MP intake was 32.5 ± 66.8 mg in the perennial group and 23.1 ± 46.0 mg in the seasonal group. MP was withdrawn in 73.9% of patients in the perennial group and in 77.8% in the seasonal group.

### 2.3. Changes in Eosinophils, FeNO, and IgE Concentration

**Figure 3:**
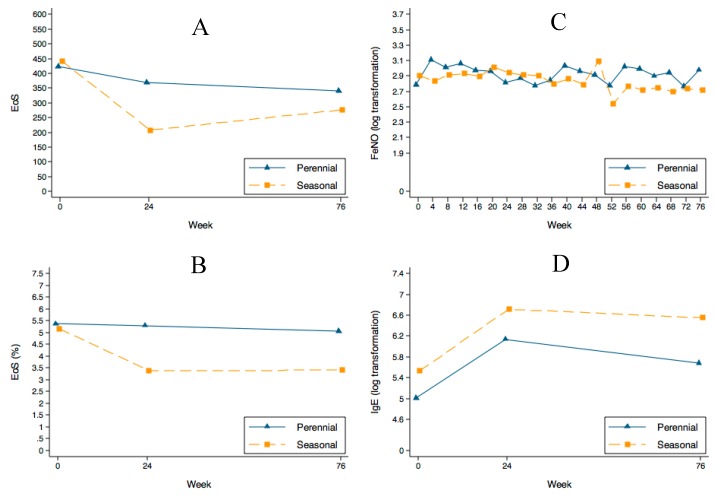
Changes in eosinophil count, FeNO and IgE values during follow-up in both groups. (**A**,**B**) show the decrease in eosinophils. A non-statistically significant trend towards a decrease in the absolute number of cells (*p* = 0.086) was found. Although the difference was not statistically significant, the seasonal group showed a more marked decrease. FeNO values (**C**) remained stable along the follow-up and the IgE concentration (**D**) initially increased in both groups—as expected—and showed a trend to stabilize or slightly decrease during follow-up.

Figure 3 shows the changes in eosinophils, FeNO, and IgE concentration. 

**Figure 4:**
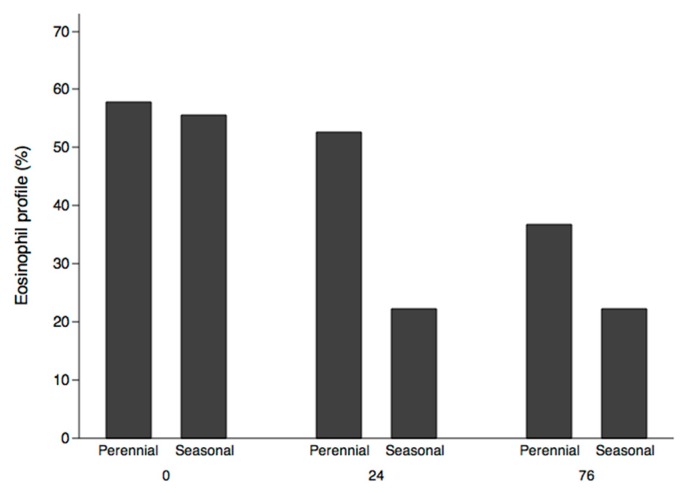
Changes in eosinophil profiles (more than 300 eosinophils/µL in peripheral blood) over the course of the study.

Eosinophils showed a trend towards a decrease in the absolute number of cells (*p* = 0.086) but not in percentage (*p* = 0.251) (Figure 3A,B). The percentage of patients with an eosinophil profile above 300 cells decreased during follow-up: in perennial allergic patients from 57.9% at entry to 52.6% at week 24 and to 36.8% at week 76 (*p* = 0.194), and in seasonal patients from 55.6% at entry to 22.2% at 24 weeks, remaining at 22.2% at 76 weeks (*p* = 0.134) (Figure 4). 

FENO median values at entry were within normal limits (Table 1). Figure 3C shows that FENO values remained stable during omalizumab treatment, despite the reduction in oral corticosteroids. 

IgE concentration increased significantly (*p* = 0.001) with a *β*_week_ = 0.030. Although the trend was similar in the two groups, the values were consistently higher in the seasonal group (*β*_seasonal_ = 0.656; *p* = 0.026) (Figure 3D).

### 2.4. PFTs Evolution

**Figure 5:**
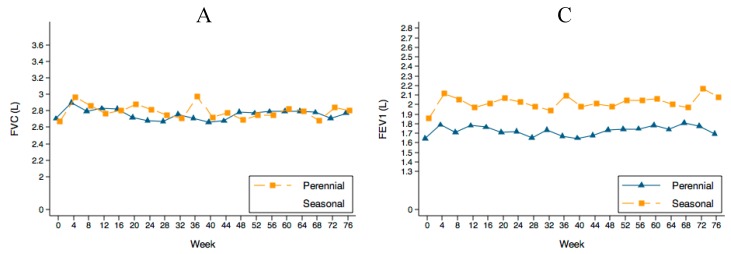

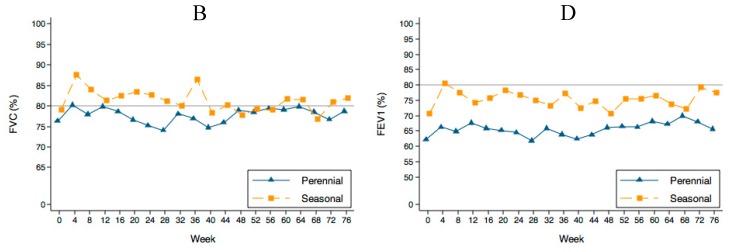
Evolution of the spirometries during follow-up. (**A**,**C**) show the absolute value in liters (L) and (**B**,**D**) the percentatge of the predicted value. The lines in (**B**,**D**) show the normal value. FVC: forced vital capacity. FEV_1_: forced expiratory volume in one second.

Despite oral MP decrease, PFTs did not deteriorate (Figure 5) in either group. However, the seasonal allergen patients maintained higher spirometry values (around 200 mL) which, although not statistically significant, could be considered clinically relevant. FEV_1_/FVC values were 60.7% ± 13.1% and 69.1% ± 12.8% for perennial and seasonal patients respectively at entry and 61.3% ± 12.2% and 73.4% ± 11.6% at the end of follow-up.

### 2.5. Side Effects

The treatment was well-tolerated and no relevant side effects were detected.

## 3. Discussion

In November 2006, the GINA [1] later followed by the Guía Española para el Manejo del Asma GEMA [11] and some other guidelines included a biological treatment for bronchial asthma for the first time. This treatment was omalizumab, which had shown its clinical efficacy and effectiveness [4,5,6,7,8,9]. This drug was marketed for severe IgE-mediated asthma patients allergic to a perennial allergen and was included as add-on therapy in GINA step V asthma patients. Some authors have proposed that omalizumab may have a wider range of possible prescriptions. Nonetheless, the manufacturer has not changed the prescription criteria for asthma. The drug is still recommended only for severe asthma patients allergic to at least one perennial allergen.

Omalizumab has been tested in cases of rhinitis caused by seasonal allergens causing symptoms during the season of pollination, with great success [12] as well as in perennial allergic rhinitis [13]. Our purpose in the present study was to evaluate whether omalizumab could be as effective in severe oral corticosteroid-dependent chronic asthma attributed to a seasonal allergen as in patients affected by a perennial allergen. 

We were careful to identify seasonal allergic patients as reliably as possible in order to distinguish them from perennial allergen patients. We recruited two patients allergic to perennial allergens for every patient with seasonal allergy. This procedure was chosen to allow a better comparison of the behavior of the perennial group with that of our previously published cohort [14], which included 32 patients followed up during two years. All patients were oral corticosteroid-dependent and fulfilled the criteria for omalizumab treatment, except for the fact that one group included sensitization to seasonal allergens. The rate of non-responders, between 5% and 10%, was in accordance with our previous experience [14]. The patient sample size was calculated based on our empiric experience since no previous information was available. We considered a minimum of 10 patients to be representative if the behavior of the group was homogeneous. Our results showed that both groups had a similar clinical response to omalizumab.

In the past few years, several attempts have been made to categorize severe asthma into phenotypes by the application of unsupervised cluster analyses. Apart from allergic asthma, the most prominent phenotype to emerge is characterized by eosinophilic airway inflammation in spite of using high-intensity anti-inflammatory treatment [15,16]. About one-third of patients with severe asthma are considered to have this refractory eosinophilic asthma phenotype [17]. Our cohort of patients could be included in this group, since all were receiving oral corticosteroids but had a notably high concentration of eosinophils (> 400 cells and > 5%); these rates were above the ones used in the DREAM [16] and MENSA [17] studies for defining patients as eosinophilic and thus eligible for prescription of an anti-IL5 mAb [18]. In the MENSA study, the geometric mean of eosinophils was 295 cells per microliter [19]. In both our groups, the eosinophil count fell progressively after omalizumab exposure, though the reduction was more marked after 24 weeks in the seasonal group. Moreover, the percentage of patients showing the eosinophil profile also decreased notably and with a similar intensity in both groups. This is not surprising because in the airways of patients with allergic asthma, the drug reduces FcεRI+ (IgE high affinity receptor) and IgE+ cells and causes a profound reduction in tissue eosinophilia, together with reductions in submucosal T-cell and B-cell counts [5,20].

FeNO values were mainly normal or quite low, in any case lower than the values found in the DREAM [18] and MENSA [19] studies. This is probably because all patients were oral corticosteroid-dependent. The number of overweight or obese patients was also similar, thus ruling out excess weight as a potential confounding factor.

The behavior of the two groups was quite similar and no statistically significant intergroup differences were found. The decrease in the year exacerbation rate in both groups despite the decrease in the oral corticosteroid (OC) consumption endorses the clinical effect of omalizumab toward a better disease stabilization and control. The only sustained difference was the permanently higher FEV_1_ value in the seasonal group that, though not statistically significant, was clinically relevant (200 mL at entry and maintained during the follow-up). This suggests that permanent exposure to allergens may be more harmful than episodic exposure. The side-effect profile was highly favorable in both groups.

The study has several limitations, the most important being the reduced number of patients and the lack of a placebo randomized control group. Rather than comparing two different treatments, we assessed the clinical response to the same treatment in two different populations: patients allergic to perennial allergens and patients allergic to seasonal allergens. The only way to perform a randomized study would be to have had a treated and a placebo arm in each group. Considering the impossibility of preparing a reliable placebo, and the fact that omalizumab is indicated only for patients allergic to at least one perennial allergen, a study of this kind would have to be done by the pharmaceutical company. Thus, we believe that our study represents the only way to make this information available in the literature. Finally, distinguishing between perennial and seasonal patients is extremely complex although we are confident that we were able to do so. Our discussion, based on our results helps to make understandable why chronic severe allergic asthma patients sensitized to seasonal allergens can also benefit from anti-IgE treatment. Since it has been shown in previous studies that sensitization to different allergens is not associated with significant differences in severity and control of asthma [21] one could expect similar results regarding treatment.

The results of several large randomized trials have shown that omalizumab is effective and well-tolerated as an add-on therapy in patients with severe persistent allergic asthma due to perennial allergen exposure. Omalizumab treatment significantly improves symptoms and disease control, reduces asthma exacerbations, and increases patients’ quality of life [22]. Our study corroborates these findings, but also shows that omalizumab is helpful for severe chronic asthma caused by seasonal allergens.

## 4. Materials and Methods

### 4.1. Study Design

In this prospective interventional study performed at the Corporació Sanitària Parc Taulí, a 750-bed university hospital with a reference area of 450,000 inhabitants, two groups of patients with uncontrolled severe oral corticosteroid dependent allergic asthma were treated with the same drug, omalizumab. The study was approved by the hospital’s ethics committee (CEIC/CEIm Institut d'Investigació i Innovació Parc Taulí-2017/527). Since the treatment for asthma attributed to a seasonal allergen was an off-label indication, its use was considered as compassionate; in accordance with the Spanish legislation, written informed consent was obtained in these patients.

### 4.2. Population

Eligible patients met the ERS/ATS criteria for a diagnosis of severe asthma; all had stable treatment requirements of at least 1000 µg fluticasone propionate with 100 µg of salmeterol or equivalent. Patients were considered to be corticosteroid-dependent when they required a minimum of 4 mg of methyl-prednisolone (MP) per day ≥1 year or boosters of MP equivalent to a mean daily dose of 4 mg of MP. Participants had to maintain their treatment (standard of care) throughout the study. Exclusion criteria included past or present smoking, substantial uncontrolled comorbidity, possibility of pregnancy, and history of poor treatment adherence. 

Uncontrolled asthma was defined according to the ERS/ATS criteria [23]. Patients had to follow any one of the following criteria: frequent severe exacerbations (two or more bursts of systemic oral corticosteroids (OCs) (>3 days each) in the previous year or requiring to double their baseline dose of OCs); serious exacerbations: at least one hospitalization, Intensive Care Unit stay or mechanical ventilation in the previous year; airflow limitation: FEV_1_ < 80% predicted (in the presence of reduced FEV_1_/FVC defined as less than the lower limit) following a withhold of both short-and long-acting bronchodilators.

We classified patients into two groups: (a) those with asthma due to seasonal allergens, and (b) those with asthma due to perennial allergens. In both situations, a cause-effect relationship had to be clinically established. For each patient with asthma due to seasonal allergens, we recruited the next two consecutive patients with asthma due to perennial allergens. The criteria used to discern the type of allergen causing the asthma were the following: (a) when a single perennial allergen was involved (dust mites or moulds), the asthma was considered to be caused by a perennial allergen; (b) When the single allergen was a pollen, and the hospital patient’s folder revision revealed that the asthma showed seasonal symptoms at the beginning, the asthma was considered seasonal; (c) in cases in which both seasonal and perennial allergen sensitization was detected, in patients sensitized to epithelia at the time of the first skin prick tests, we made all possible efforts to ensure that exposure was stopped. In addition to the clinical information at the hospital we reviewed the electronic medical records of patients in which the primary care physician had documented the absence of animal exposure. In case of doubt, we personally contacted the patient's primary care physician. Thus, although persisting, this sensitization was considered irrelevant to disease’s evolution. All patients with pollen seasonal allergy had perennial symptoms before the appearance of house dust mite sensitization. 

Non-responders were those patients in which omalizumab did not allow a decrease in the number of exacerbations and/or oral corticosteroid consumption.

The primary outcomes were the oral corticosteroid-sparing effect of omalizumab and the rate of exacerbations. The secondary variables were changes in pulmonary function tests (PFTs) and the exhaled fraction of nitric oxide (FeNO). The safety outcome was the reporting of side effects. 

### 4.3. Size Calculation

We planned to recruit a minimum of ten patients in the seasonal group. For each patient with asthma due to a seasonal allergen, we recruited the next two consecutive patients with asthma due to a perennial allergen that fulfilled the criteria. We assumed a loss of 10% in each group.

### 4.4. Procedures

(a) A skin prick test was performed in every patient (ALK-Abello^®^ testing allergens); (b) The test was considered positive when the reaction was ≥ to histamine and ≥3 mm; (c) Omalizumab dose was calculated according to the Novartis^®^ dosing table based on IgE concentration and patient's weight. Depending on the total dose, the drug was administered subcutaneously, either bi-weekly or monthly; (d) Patients were considered responders or non-responders after 24 weeks of omalizumab treatment; (e) The dose of oral MP was tapered at a rate of 2 mg every two weeks depending on tolerance, as in previous studies [4,14]; (f) At each monthly visit, a forced spirometry and FeNO measurement were performed and the accumulated monthly MP dose was calculated; (g) The follow-up lasted 76 weeks; (h) Side-effects were investigated at each outpatient visit.

### 4.5. Statistical Analysis

Descriptive analysis was performed using central tendency and dispersion measures for continuous variables and the distribution of frequencies for categorical variables. Depending on the variable distribution, *t*-test, Wilcoxon rank-sum, or Fisher’s exact test was used to analyze the homogeneity between groups. Linear mixed-models with two levels (subject and visit) were fitted to analyze the evolution of the different parameters of interest over time. Because the distributions of residuals of corticoids, FeNO and IgE were skewed, data were log-transformed for analysis. In all cases, a *p* score less than 0.05 was considered statistically significant. Software STATA version 11.2 (Stata Corp., College Station, TX, USA) was used.

## 5. Conclusions

These findings have two direct implications, one pathophysiological and one clinical. From the pathophysiological point of view, we can now confirm that it is the presence of the IgE that causes the persistence of the allergic cascade, and that allergens are merely a trigger factor. This would at least be proven in case of asthma due to seasonal allergens. In case of perennial allergens, the continuous exposure to the allergen precludes us extending this conclusion to this group. The clinical implication is that omalizumab prescription should be extended to severe asthma patients allergic to seasonal allergens.

## Figures and Tables

**Table 1:** Demographic data at entry of the whole group and the two subgroups.

	All		Perennial		Seasonal	
	*n*	%	*n*	%	*n*	%
Male/Female	9/19	32.1/67.9	8/19	42.1/57.9	1/9	11.1/88.9
	**Median**	**IQR**	**Median**	**IQR**	**Median**	**IQR**
Exacerbation (year/rate)	2.67	3.17	2.67	2.67	2.67	4.00
Omalizumab dose	300	450	300	300	300	300
Corticoids	120	224	120	224	120	168
IgE	178.5	254.8	138	173.1	229	184
FeNO	15	32	14	25	17	26
	**Mean**	**SD**	**Mean**	**SD**	**Mean**	**SD**
Body Mass Index	27.43	5.75	27.68	4.25	26.91	8.39
EoS (%)	5.31	3.53	5.2	3.79	5.29	2.93
EoS	429.29	313.83	410.00	328.67	440.00	274.10
FVC	2.69	0.78	2.71	0.90	2.67	0.52
FVC%	77.21	17.99	76.32	20.38	79.11	12.35
FEV_1_	1.71	0.59	1.65	0.63	1.85	0.49
FEV_1_%	64.93	20.66	62.21	22.29	70.67	16.36
FEV_1_/FVC%	63.43	13.34	60.74	13.08	69.11	12.75

FeNO: fraction exhaled of nitric oxide. EoS: eosinophils; FEV_1_: forced expiratory volume in one second; FVC: forced vital capacity; IQR: interquartile range; *p*-value was not significant for all the variables compared.

**Table 2:** Allergen detected by skin prick test.

Perennial allergens (*n* = 20)		Seasonal allergens (*n* = 10)	
House dust mites	13	Grass pollens	2
Moulds	2	Grass Pollens + cat epithelium	1
House dust mites + cat and dog epithelium + grass pollen	2	Cupressus Pollens + cat epithelium	1
House dust mites + grass pollen	1	Grass Pollens + cat and dog epithelium	2
House dust mites + cat epithelium + moulds	1	Grass Pollen + bird feathers	1
House dust mites + cat epithelium + grass pollen + moulds	1	Grass Pollen + rabbit epithelium	1
		Grass Pollen + house dust mites	1
		Parietaria Pollen + house dust mites	1
